# Economic attributes and childhood stunting in Rwanda: case study of the City of Kigali

**DOI:** 10.11604/pamj.2022.42.157.30650

**Published:** 2022-06-28

**Authors:** Manassé Nzayirambaho, Aimable Nsabimana, Vincent Manirakiza, Pierre Claver Rutayisire, Kato Njunwa

**Affiliations:** 1 School of Public Health, College of Medicine and Health Sciences, University of Rwanda, Kigali, Rwanda,; 2 Department of Economics, College of Business and Economics, University of Rwanda, Kigali, Rwanda,; 3 College of Education, Geography, University of Rwanda, Kigali, Rwanda,; 4 Department of Applied Statistics, College of Business and Economics, University of Rwanda, Kigali, Rwanda,; 5 School of Health Sciences, College of Medicine and Health Sciences, University of Rwanda, Kigali, Rwanda

**Keywords:** Economic attributes, stunting, under five children, Kigali, Rwanda

## Abstract

**Introduction:**

stunting in under five children is a great concern in low and middle-income countries including Rwanda. While over the past decades different developing countries have made remarkable efforts improving their economic growth, there is mixed evidence and lack of consensus on the impact of economic development on nutrition improvement. The objective of this study was to assess the relationship between economic attributes and childhood stunting in the City of Kigali.

**Methods:**

this was a retrospective cross-sectional and comparative study documenting the period 2010-2017. Stunting in under five children was analyzed in relation to the economic attributes which include the household consumption per capita, annual household income and level of poverty. The analysis was done at the level of district. Official reports from the National Institute of Statistics of Rwanda provided data on both economic attributes and stunting.

**Results:**

in some situations, the improvements in economic attributes such as increase in average household consumption per capita and increase in annual household income are followed by the reduction of stunting in under five children. However, in some other situations, the reduction of the level of poverty and the increase of annual household income was not translated into the reduction of stunting.

**Conclusion:**

improvements in some economic attributes do not necessarily translate into reduction of stunting in under five children. Further studies are needed to understand possible lead forces underlying this situation including establishing the proportion of household income spent on children´s nutrition as well as possible inequity and inequality in wealth distribution.

## Introduction

Undernutrition and child mortality remain persistent obstacles to economic and human development in Africa [1-4]. Studies have shown that chronic undernutrition is a crucial predictor of child morbidity and mortality, and have long-term consequences on intellectual development in which adversely affect academic and professional performance, thereby reducing the chances for personal advancement [5, 6]. While important gains have been reported in terms of fighting poverty and improving access to water and sanitation, electricity, school enrolment, and food security, which constitute a prerequisite for improved nutritional outcomes for children [7, 8], sub-Saharan Africa and South Asian countries still account for most of the childhood undernutrition globally. Indirectly, this situation contributes to poor economic and human development outcomes in these countries [4, 7].

It is in this context that particular attention has been paid to the role of childhood growth restriction and stunting in low and middle-income countries [9] and this was reflected in the indicators on child undernutrition not only in the Millennium Development Goals [10], but also in the post-2015 Sustainable Development Goals [11, 12]. Over the past two decades, the relationship between economic growth and child nutrition has remarkably attracted attention of researchers and development partners [13-16]. From the data of 74 developing countries recorded between 1984 and 2014, it was observed that economic growth is not a sufficient condition for a reduction in child undernutrition [17]. For example, this was the case in South Asia, where rates of childhood stunting remain at 38% in spite of rapid economic growth in some countries like India [18].

A recent study conducted in Ghana, Kenya and Zambia concluded that “the cumulative effects of economic growth, poverty reduction and other polices to improve health and nutrition appear to have been translated into reductions in stunting rates. However, inequalities have persisted and seem to be widening over time. The poor and those living in rural areas are the most affected” [19]. Economic growth can contribute to worsening inequalities in a different manner, including access to education, health and technology, and growth can reduce poverty only if the distribution of income remains constant over time [15]. Analyzing the relationship between childhood stunting and economic outcomes in adulthood, a recent review indicated that economic growth is effective in reduction of stunting when “increases in national income are directed at improving the diets of children, addressing gender inequalities and strengthening the status of women, improving sanitation and reducing poverty and inequities” [20]. In addition, full immunization, iron supplements and deworming medication have been suggested as factors that can contribute better to the reduction of childhood stunting [21].

In some developing countries, the problem of stunting is aggravated by the fast-changing demography, labor market and sociocultural environment. In their fight for a better life, a growing number of people leave the agricultural sector and migrate to urban regions in the hope of having better jobs and future prospects [22]. The same applies to Rwanda where the City of Kigali is the second main destination for recent internal migration after the Eastern Province. Nearly one third (29%) of recent migrants headed to Kigali, with the districts of Gasabo and Kicukiro being especially popular destinations [23].

The demographic growth and migration to urban areas is considered to be the main factor that contributes to the Rwandan rapid urbanization. Indeed, the urban population which was 16.5% in 2012 was expected to rise to 35% by 2020. The increasing rural to urban migration towards the City of Kigali and other Secondary Cities is challenging the supply and access to basic amenities for livelihood, such as existing infrastructure for education and health services. Internal migration is, therefore, expected to display a close impact on socio-economic status, education and health outcomes of households at place of origin as well as at place of destinations, and this link is even more pronounced in households with children [24].

The same situation is observed in other countries where the increasing trend of rural to-urban migration is leading to serious labor imbalances, increasing urban poverty and raising pressure on the weak healthcare infrastructure, as well as aggravating the situation of health and nutrition among urban population [25]. Different studies have shown that in such a situation, childhood nutritional status is an excellent outcome indicator because it is closely linked to poverty and it reflects the general level of deprivation and inequalities in development [26]. Poor nutritional status during childhood has both immediate and long-term consequences. It is incriminated in more than half of all child mortality [27, 28].

Stunting, which is the percentage of children with low height-for-age, also commonly referred to as chronic under nutrition, is considered to be the indicator of choice to analyze the relationship between under nutrition and poverty. “It reflects poor linear growth caused by sustained food deprivation, repeated illness or both. Stunting is considered a barometer of the population´s ability to meet basic needs, such as food, health care and housing” [26]. Stunted children develop weakened immune systems and become more susceptible to diarrhea and other infections. Evidence also suggest that children suffering from malnutrition in early life have decreased work capacity in adolescence, compared with their counterparts who had normal nutritional status during early childhood [29].

However, “there is mixed evidence and lack of consensus on the impact of economic development on nutrition improvement, and there is a dearth of empirical studies on this relationship in the case of sub-Saharan Africa” [30]. The objective of the present study, therefore, was to assess the relationship between economic attributes and childhood stunting in the City of Kigali. The study used only data extracted from different official sources. The main rationale was to contribute to the current evidence base and assist in policymaking for child nutrition-related programmes.

## Methods

**Study design and variables:** this was a retrospective cross-sectional and comparative study documenting the period 2010-2017. Recent national surveys and relevant reports as well as publications have been extensively desk reviewed on the topic. Stunting in under five children was analyzed in relation to the economic attributes which include the household consumption per capita, annual household income and level of poverty. The analysis was done by comparison of data at the level of district.

**Figure 1:**
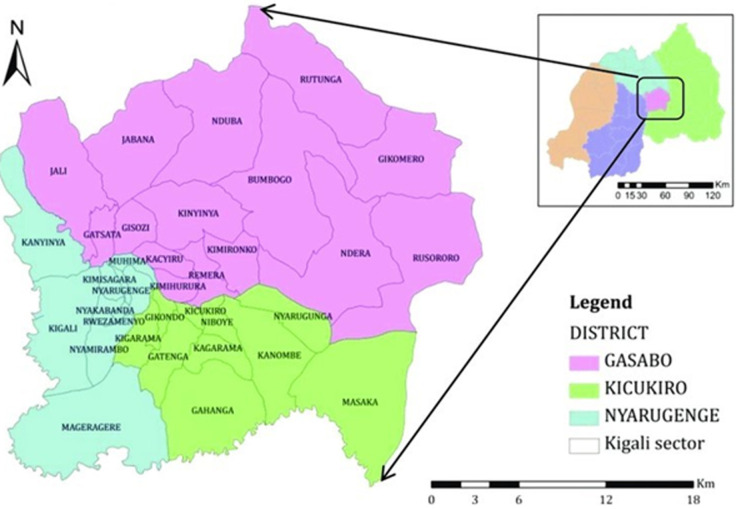
administrative map of the City of Kigali

**Setting:** the city of Kigali (CoK) is the capital and the largest city of Rwanda. It spreads on 730 km^2^ with a population of around 1.6 million [31]. It is divided into three (3) districts, namely Nyarugenge, Kicukiro and Gasabo. Each district is subdivided into sectors, cells and villages. Overall, in the CoK there are 35 sectors, 161 cells, and 1061 villages. Nyarugenge district is located in the eastern part of the city and it covers an area of 134 km^2^. It is comprised of 10 sectors, 47 cells and 232 villages. Kicukiro district has an area of 166.7 km^2^ and is located in the southern part. It has 10 sectors, 41 cells and 328 villages. Gasabo district which is the largest with 430 km^2^ comprises 15 sectors, 73 cells and 501 villages [31] (Figure 1).

**Figure 2:**
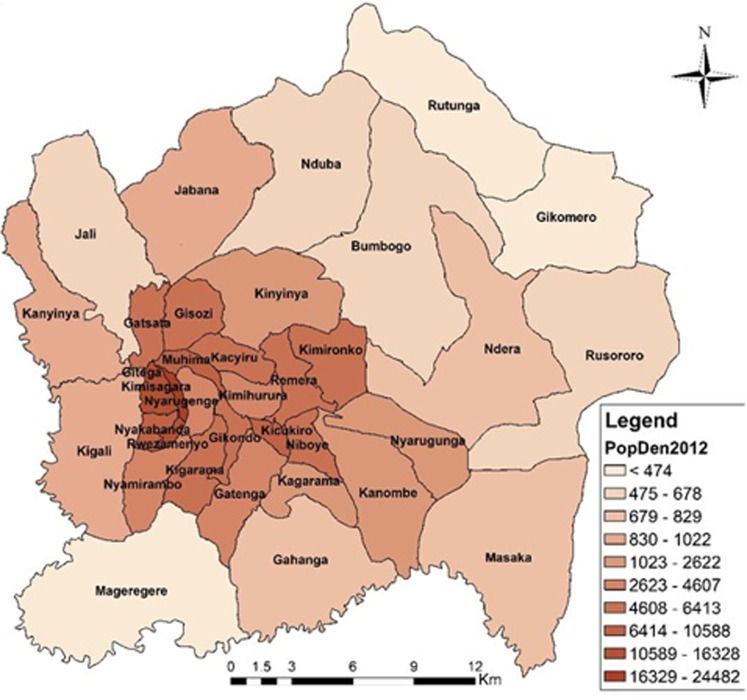
population density of the City of Kigali by sectors in 2012

The level and the process of urbanization among the districts is heterogeneous with the population density and variety of businesses and infrastructures decreasing from the Central Business District (CBD) to the peripheral sectors. The CBD is located in Nyarugenge around which emerged the first settlements of Kigali. Currently Nyarugenge has the most densely populated sectors, namely Gitega, Kimisagara, Rwezamenyo and Muhima (10,000 to 20,000 inhabitants per km^2^). Entire or larger portions of some sectors of the CoK are still characteristically rural, like Rutunga and Gikomero of Gasabo district, and Mageragere in Nyarugenge with the density of less than 500 inhabitants km^2^. Likewise, large parts of Kinyinya, Jali, Jabana, Nduba, Bumbogo, Ndera, and Rusororo in Gasabo district still show rural features. This is the same for Masaka, Nyarugunga, Kanombe and Gahanga in Kicukiro district, as it is with Kigali and Kanyinya in Nyarugenge district (Figure 2).

Habitat mosaic and access to basic services also reflect the urban development process. Many old and unplanned neighborhoods are located in the inner city area in Nyarugenge. Throughout the urban expansion, new planned and modern neighborhoods emerged in Gasabo and Kicukiro districts enabling the two districts to show appreciable urban development indicators than Nyarugenge. Over 42 planned neighborhoods identified in Kigali, Nyarugenge account for only 7 against 13 for Kicukiro and 22 for Gasabo. Over 31 residential neighborhoods identified in Nyarugenge, about a half are unplanned, seven are planned and nine mixed or semi-planned. With regard to the adequacy of housing, the City of Kigali stands at 40%. High improvement is in Kicukiro district whereby 50% of households live in adequate housing, 39% for Gasabo and 30% for Nyarugenge [32].

**Data sources and measurement:** the reports from the National Institute of Statistics of Rwanda (NISR), especially the three consecutive rounds of Integrated Household Living Conditions Survey of Rwanda (IHLCS), in French commonly known as *Enquête Intégrale sur les Conditions de Vie des ménages*, EICV (EICV3, EICV4 & EICV5) conducted in 2010/11, 2013/2014 and 2016/2017 provided data on economic attributes while the Rwanda Demographic and Health Survey (RDHS) of 2010 and 2014-2015 provided data on stunting in under five children. By the time the manuscript of this article was written, the final report of RDHS 2019-20 was not yet published.

**Demographic and health surveys:** the RDHS 2010 and 2014-15 were implemented by the National Institute of Statistics of Rwanda and the Ministry of Health. ICF International provided technical assistance for the survey through the DHS program, a USAID-funded project providing support and technical assistance in the implementation of population and health surveys in countries worldwide. The RDHS 2010 and 2014-15 samples were nationally representative household-based surveys, with a two-stage sample design. The first stage involved selecting sample points (clusters) consisting of enumeration areas (EAs) also known as villages. A total of 492 clusters were selected in 2010 and 2014-15. The second stage involved systematic sampling of households. A household listing operation was undertaken in all of the selected EAs and households to be included in the surveys were randomly selected from these lists. Twenty-six households were selected from each sample point, for a total sample size of 12,792 households in 2010 and 12,793 households in 2014-15.

The anthropometric component for children under the age of 5 years was done in a subsample of 50 percent of the households. Height measurements were carried out using a Shorr measuring board also produced under the guidance of UNICEF. Children younger than age 24 months were measured lying down (recumbent length) on the board, whereas standing height was measured for older children. Based on these measurements, height - for -age index was constructed for child stunting. Children whose height-for-age Z-score was below minus two standard deviations (-2 SD) from the mean of the reference population were considered short for their age (stunted) and chronically malnourished.

The child stunting indicator was calculated using growth standards published by the World Health Organization (WHO) in 2006. These growth standards were generated through data collected in the WHO Multicentre Growth Reference Study [33]. The WHO child growth standards can be used to assess children all over the world, regardless of ethnicity, social and economic influences, and feeding practices. The standards replaced the previously used reference standards of the U.S. National Center for Health Statistics, accepted by the U.S. Centers for Disease Control and Prevention (NCHS/CDC/WHO) in 1977. Data collections were conducted from September 26, 2010 to March 10, 2011 and from November 9 to April 8, 2015 respectively for RDHS 2010 and RDHS 2014-15 [34, 35].

**Integrated Household Living Conditions Surveys (EICVs):** the EICVs are nationally representative, repeated cross-sectional surveys conducted about every five years starting in 2000. Each of the surveys lasted for 12 months. The 2010 survey commenced in November 2010 and finished in October 2011, the 2014 survey conducted from October 2013 to October 2014, and the 2016 survey from October 2016 to October 2017. There were 14,308, 12,312 and 14,580 households in the 2010, 2014 and 2016 EICV, respectively, with households being selected based on a stratified two-stage sample design. Sample villages were selected within each stratum (district) systematically with probability proportional to size at the first sampling stage and sample households were selected from each sample village at the second stage [36-38]. From the three EICVs, data on three economic attributes were considered in this research: household consumption per capita, annual household income and the level of poverty.

## Results

**Participants:** in 2010, countrywide, a total of 4,356 children under age 5 were eligible to be measured for weight and height and had complete and valid anthropometric data collected including 397 children for the City of Kigali (104 children for Nyarugenge district, 175 children for Gasabo district and 118 children for Kicukiro district). Likewise, in 2014-15, a total of 3,884 children under age 5 were eligible to be measured for weight and height, and 3,813 (97 percent) including 419 children from the City of Kigali (Nyarugenge district 113, Gasabo district 203 and Kicukiro district 103) had complete and valid anthropometric data collected.

### Main results

**Economic attributes in the City of Kigali:** Table 1 shows the distribution of the three economic attributes in all districts of the CoK, across the three rounds of the EICVs (2010-11, 2013-14 and 2016-17) (Table 1).

**Table 1:** economic attributes in the City of Kigali

District	EICV 2010-11	EICV 2013-14	EICV 2016-17	Overall change (2011 & 2017) (%)
**Average household consumption per capita (in Rwf)**				
Gasabo	1,146,147	1,150,268	1,295,603	13%
Kicukiro	1,150,268	1,195,167	1,378,369	19.8
Nyarugenge	1,242,245	996,115	1,199,316	-3.4
Overall city of Kigali	1,179,553	1,113,850	1,291,096	9.5
**Annual household income (in Rwf)**				
Gasabo	3,657,558	3,847,658	4,544,633	24.3
Kicukiro	4,411,721	5,312,814	6,096,470	38.2
Nyarugenge	4,130,796	4,183,387	5,135,567	24.3
Overall city of Kigali	4,066,692	4,447,953	5,258,890	29.3
**Level of Poverty (in %)**				
Gasabo	13.2	7.5	5.0	-8.2
Kicukiro	10.9	10.8	6.4	-4.5
Nyarugenge	8.6	9.4	1.0	-7.6
Overall city of Kigali	10.8	9.4	4.3	-6.5

**Household consumption per capita:** our results reveal that between the periods of 2011 and 2017, while the annual household consumption per capita in Gasabo and Kicukiro districts increased to 11 and 16 percent respectively, the consumption per capita in Nyarugenge relatively declined with an average of 3 percent [36-38]. Furthermore, Table 1 indicates that in recent years, Kicukiro has been the wealthiest urban area district in the CoK, with an annual average household consumption per capita of about 1.38 million Rwandan francs, which is equivalent to US dollars 1500 per year per person. This indicates that the district a higher consumption per capita than that at national level which is still around US dollars 720 per person per year. In general, the findings show that over the last 8 years, the consumption level of Kigali city dwellers has increased by 8 percent on average. However, this trend is relatively smaller in Nyarugenge district.

**Annual household income:** there is a progressive increase in annual household income for all the districts, over the three survey cycles. Between 2011 and 2017, the annual household income in Nyarugenge, Kicukiro, and Gasabo districts underwent a significant increase of 24.3, 38.2 and 24.3%, respectively, while the annual household income in CoK has increased by an average of 29.3%. On this attribute, Kicukiro is again the wealthiest district in CoK.

**Level of poverty:** between 2011 and 2017, there was a significant decrease in the level of poverty across all the districts of CoK (Table 1), where the decrease was an average of 8.2%, 4.5% and 7.6% in Gasabo, Kicukiro and Nyarugenge districts, respectively. In 2017, Nyarugenge district experienced the lowest level of poverty as compared to the remaining districts of CoK while Kicukiro had the highest level of poverty.

**Childhood stunting in the City of Kigali:** Table 2 shows the distribution of stunting in under five children for the period of 2010-2015 in the City of Kigali (Table 2).

**Table 2:** stunting in under five children in the City of Kigali (in %)

District	Rwanda demographic and health survey 2010	Rwanda demographic and health survey 2015
Gasabo	23.8	22
Kicukiro	18.9	17
Nyarugenge	28.3	29
Overall city of Kigali	23.5	23

**Stunting in under 5 children:** at the Kigali City level, 23% of children under 5 years of age were stunted (too short for their age) in 2015, while this percentage was 23.5 in 2010. In 2010 and 2015, variation by district is quite evident, with stunting being highest in Nyarugenge, followed by Gasabo and lowest in Kicukiro. There is a slight reduction of stunting between 2010 and 2015 in Gasabo and Kicukiro districts, while it increased from 28.3% to 29% in Nyarugenge district.

## Discussion

The study discusses the relationship between socio-economic attributes and stunting in under five children across the three districts of the City of Kigali. By looking at Table 1 and Table 2, we find that between 2011 and 2017, Nyarugenge district had the lowest household consumption per capita (Rwf 996,115 in 2014 and Rwf1, 199,316 in 2017) among the three districts of the City of Kigali, while it had the highest levels of stunting among children aged under five years in 2010 and 2015 (28.3% and 29% respectively). On the other hand, the results show that between 2011 and 2017, Kicukiro district had the highest household consumption per capita (Rwf 1,195,167 in 2014 and Rwf1,378,369 in 2017) among the three districts of the City of Kigali while it had the lowest levels of stunting in under five-year-old children in 2010 and 2015 (18.9% and 17% respectively). This indicates an inverse relationship between the level of household consumption and the level of stunting in children under age five. The implication of this is that in the City of Kigali, as households increase their consumption, their diets are also improved, enabling them to ensure the nutrition of their children. This is consistent with the study of Torlesse *et al*. [39] who found that as households are enabled to spend more on food, they improve the quality and quantity of their diets, thereby sustain the nutrition of their children.

The present study also discusses the association between the level of annual household income and the level of stunting in children under five years old. The findings in Table 1 indicate that, from 2011 to 2017, on average, Gasabo district had the lowest level of annual household income (Rwf 3,847,658 in 2014 and Rwf 4,544,633 in 2017) among the three districts of Kigali city, followed by Nyarugenge district (Rwf 4,183,387 in 2014 and Rwf 5,135,567 in 2017), whilst Kicukiro district was the wealthiest (Rwf 5,312,814 in 2014 and Rwf 6,096,470 in 2017) among the three districts. Conversely, the findings indicate that in 2010 and 2015, the percentage of stunted children aged under five years was the highest in Nyarugenge district, followed by Gasabo, whilst Kicukiro district had the lowest percentage of stunting in children under the age five years. This highlights the average difference of about 12% between the latter district and Nyarugenge. The greater percentage of stunting in Nyarugenge district in comparison with Gasabo district (a difference of 7%) despite its higher level of household income, may be attributed to the low share of income assigned to household consumption, especially to food consumption in Nyarugenge district as compared to Gasabo district, which may limit their access to high nutritious diets. It may also be due to the fact that food prices in Nyarugenge are higher than in Gasabo districts, given that Gasabo district has many rural areas with more agricultural activities, hence has more nutritious food supply than Nyarugenge district.

In some other studies, increases in household incomes and reduction in poverty levels have been linked to a higher caloric consumption [40]. Different authors have confirmed that a low family income and poor living conditions increase the risk of child stunting, as a result of high food insecurity, low access to health care, unhealthy environments and a high risk of infections [41, 42]. A study conducted in five countries (Lebanon, Morocco, Syrian Arab Republic, Tunisia and Yemen), has found that the percentage of child stunting between 1991 and 2001 was always higher in the lower-income households compared to higher-income households [43]. However, it is worth to note the multifactorial nature of child stunting which include the identification of protective factors like household wealth, maternal education and the body mass index of children [44].

Looking at our findings in Nyarugenge district, it is also possible that the aggregated statistics on economic growth mask inequalities between groups. For example, if the reported increase in annual household income was observed only among a handful of people, this would explain why stunting didn´t reduce in this district. According to Coretta *et al*. [19], despite increase of national incomes, many people don´t necessarily participate in economic opportunities created and so there is not any improvement in their socio-economic status. As a consequence, together with their children, they remain at risk of malnutrition. Therefore, with increased economic growth, concerns around inequity and inequality must always be considered.

Furthermore, the present study discusses the relationship between the level of poverty and stunting among the three districts of the City of Kigali. Table 1 shows that between 2011 and 2017, on average, Nyarugenge district had the lowest level of poverty (1.0% in 2017), followed by Gasabo district (5.0% in 2017), while Kicukiro district had the highest level of poverty (6.4% in 2017). Surprisingly, Table 2 indicates that in 2010 and 2015, of the three districts, Nyarugenge had the highest levels of stunting in under five year old children, followed by Gasabo district, whereas Kicukiro district had the lowest level. This high level of stunting in Nyarugenge district despite the lowest level of poverty may be due to the fact that a large part of households in Nyarugenge are living on small wages and small earnings from small and medium enterprises (SMEs) as compared to those in the other two districts. This is consistent with the study of Kirk *et al*. [45] who found a positive link between small waged employments and stunting in children under age five. Besides, it is possible that those involved in agriculture (households living in rural areas of Nyarugenge) are more market oriented despite their limited food production, which may hinder their ability to have balanced diets, hence having adverse impact on the health of their children.

Further, the literature has also documented the direct association between child nutrition and agricultural farm input use and the weather patterns especially in developing countries where both rural and urban household livelihood depends enormously on agriculture [46, 47]. The highest level of stunting in Nyarugenge district despite the lowest level of poverty could also be explained by possible inequalities in wealth distribution as already discussed above on annual household income. Though this hypothesis would be confirmed by further studies, it supports the ideas of other authors on the matter [19].

In summary, with regard to the relationship between the improvements of economic attributes and stunting in under five children, we have two different situations: On one hand, in Kicukiro district for example, the improvements in economic attributes such as increase in average household consumption per capita and increase in annual household income are followed by the reduction of stunting in under five children. However, on the other hand, in Nyarugenge district for example, the improvements on economic attributes such as the reduction of the level of poverty and the increase in annual household income was not translated into the reduction of stunting in under five children. It is worth noting that this study used exclusively official published data aggregated at the level of district. It is therefore possible that some details would have escaped us, thereby not allowing a more in-depth analysis down to the household level.

## Conclusion

In the City of Kigali, the improvements in some economic attributes such as increase in annual household income and reduction of the level of poverty do not necessarily translate into reduction of stunting in under five children. Further studies are needed to understand possible factors underlying this situation including the use of household income on children´s nutrition as well as possible inequity and inequality in wealth distribution among households of a given district. This would allow policy makers and implementers to design more appropriate strategies while fighting against childhood stunting.

### What is known about this topic

- Childhood stunting is a major concern in developing countries;
- Undernutrition is among the main causes of childhood stunting;
- Economic growth is not a sufficient condition for a reduction in child under nutrition.

### What this study adds

- The increase in household consumption per capita is followed by reduction in childhood stunting;
- The increase in annual household income and the reduction of poverty do not necessarily translate into reduction of stunting in under five children.
